# Investigation on the control of inclusions and tensile strength in Ce-treated P110-grade oil casing steel

**DOI:** 10.1038/s41598-024-65625-w

**Published:** 2024-06-27

**Authors:** Jun Wang, Linzhu Wang, Shufeng Yang, Chaoyi Chen, Junqi Li, Xiang Li

**Affiliations:** 1 School of Materials and Metallurgy, Guizhou University, Guiyang, 550025 Guizhou People’s Republic of China; 2 Guizhou Provincial University Key Laboratory of High-Performance Battery Materials, Guizhou University, Guiyang, 550025 Guizhou People’s Republic of China; 3 School of Metallurgical and Ecological Engineering, University of Science and Technology Beijing, Beijing, 100083 People’s Republic of China; 4 School of Materials and Energy Engineering, Guizhou Institute of Technology, Guiyang, People’s Republic of China

**Keywords:** Steel, Inclusions, Tensile strength, Ce-treated, Metals and alloys, Energy infrastructure

## Abstract

This research added rare Earth elements Ce to the P110-grade oil casing steel to reveal their influence on the inclusions and tensile properties. The content of cerium in the steel varied from 0 to 452 ppm. Based on the classical thermodynamic calculation, the predominance diagram of Re-containing inclusions in P110-grade steel was obtained. The evolution route of the inclusions composition with the increasing cerium content in the steel was xCaO⋅yAl_2_O_3_ → Al_2_O_3_–CeAlO_3_ → Ce_2_O_3_–CeAlO_3_ → Ce_2_O_3_–Ce_2_O_2_S → Ce_2_O_2_S, which agreed well with the thermodynamic analysis. As the cerium content at 235 ppm, the size of Ce containing inclusions has a minimal size at 2.82 μm. Suitable Ce content can modify the big-size xCaO⋅yAl_2_O_3_ inclusions into small-size Re-containing inclusions. The results demonstrate that the tensile performance of this steel can be improved as the cerium content increases from 0 to 235 ppm. However, once the cerium content exceeds 235 ppm, further increases in cerium content led to a decline in performance. The experimental results shows that the presence of large-sized Ce_2_O_2_S inclusions and the change of microstructure, will lead to the decrease in tensile performance.

## Introduction

With the gradual depletion of easily accessible oil and gas resources in recent years, coupled with the scarcity of international petroleum reserves, oil and gas extraction is gradually shifting towards remote and extreme environments^1,2^. These environments often endure immense pressures thousands of meters underground while also being exposed to corrosive media such as H_2_S and CO_2_^3–6^. Consequently, these conditions pose higher demands on the mechanical and corrosion-resistant properties of the oil well pipe steel, which serves as the conduit for transporting oil and gas^7–9^.

Extensive research has consistently shown that non-metallic inclusions in steel significantly diminish its performance^10–12^. Scorza et al.^13^ studied the ultra-high cycle fatigue behavior of AISI 4140 high-strength steel. In steels with an average inclusion size of 7 μm, fatigue cracks tended to initiate, propagate, and extend at the sites of inclusions, and the steel had a higher fatigue life with an average inclusion size of 1.5 μm. This indicated that reducing the average size of inclusions is advantageous for enhancing the fatigue performance of steel. Furthermore, non-metallic inclusions also degraded the corrosion resistance of steel. The experimental findings by Miyoshi^14^ have revealed that in high-sulfur steel, MnS inclusions play a pivotal role as the primary cause of hydrogen-induced cracking. Moreover, the disparity in elongation between sulfides and the matrix, coupled with a lower finishing rolling temperature, will expedite the initiation of cracks. Notably, Williams^15^ made a significant observation that a discernible FeS layer, with a thickness of up to 100 nm, was observed enveloping the MnS inclusions. Remarkably, this region exhibited a propensity for preferential pitting corrosion. Specifically, in carbon steel, pitting corrosion was observed to initiate adjacent to the matrix near the MnS inclusions, while in stainless steel, the initiation of pitting occurred directly from the MnS inclusions themselves.

Rare earth elements have garnered widespread attention in recent years for their pivotal role in purifying molten steel^16–18^, modifying inclusions, and enhancing solidification microstructure and performance of steel^19^. These elements have become a prominent research focus in the steel industry. Torkamani^20^ investigated the effects of rare earth addition on the properties of low-carbon cast micro-alloyed steel; these findings revealed that the inclusion of rare earth led to a transformation of MnS inclusions into smaller-sized rare earth inclusions. Tang et al.^21^ discovered that incorporating rare earth elements could elevate the self-corrosion potential of 30CrMnSiA steel following nitriding, subsequently reducing wear rate and average friction coefficient. Liu et al.^22^ observed that with an increase in cerium addition, the sulfur (S) content and oxygen (O) content would decrease. By controlling the appropriate amount of rare earth addition, it was possible to transform the larger, angular Al_2_O_3_ and MnS inclusions into approximately spherical rare earth oxides and sulfides. Furthermore, harmful elements such as As, Sb, Pb, Sn, and P could form compounds with cerium, thereby inhibiting their precipitation at grain boundaries.

In summary, rare-earth elements have demonstrated their effectiveness in modifying inclusions and improving the microstructure, enhancing steel performance. However, it should be noted that increasing rare earth addition does not necessarily result in a continuous improvement of steel properties^23–26^. The critical threshold for enhancing enhanced performance through rare earth addition may vary for different steel grades. While there is considerable of research on adding rare earth elements in steel, studies specifically focused on rare earth treatment of P110-grade oil well casing steel remain scarce. P110-grade oil well casing steel is a high-strength steel employed in deep wells reaching depths of 5–6 km; any quality issues associated with this steel can lead to significant financial losses. This study investigated the changes in inclusions characteristics upon adding different cerium contents and subsequently calculates the predominance diagram for rare earth inclusions in this system. Furthermore, by comparing the mechanical performance variations of each sample with different Ce addition levels, the most suitable Ce addition amount in P110-grade oil well casing steel can be obtained. Such findings hold significant importance for enhancing the performance of oil casing steel and providing guidance for developing rare earth-containing steels.

## Methodology

### Experimental

P110 grade casting steel billet from a steel plant was used as the matrix steel in this experiment and the chemical composition is listed in Table 1.

**Table 1:** Chemical composition of a P110 grade casing steel (wt.%).

Element	C	Si	Mn	P	S	Cu	Ni	Cr
Content	0.26	0.26	0.47	0.01	0.0015	0.048	0.018	0.589
Element	W	Mo	V	Nb	Ti	Al	Ca	B
Content	0.022	0.759	0.0884	0.03	0.002	0.029	0.0013	0.0007

**Figure 1:**
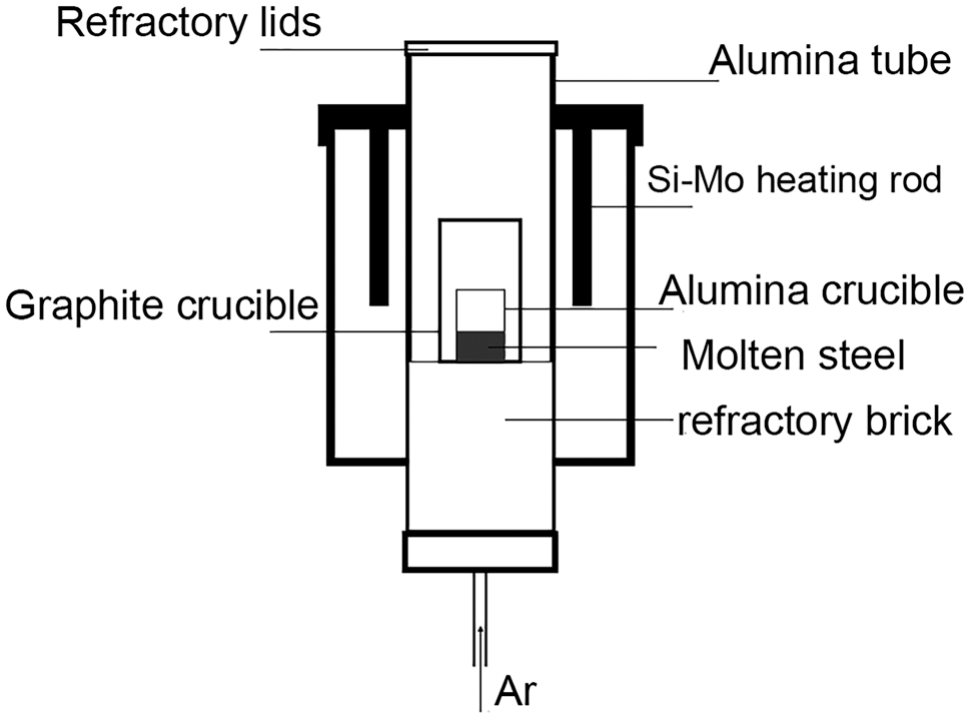
Tubular resistance furnace structure.

This experiment employed a Si-Mo high-temperature tubular resistance furnace (BLMT-1700, Luoyang, Bolaimante), as depicted in Fig. 1. The matrix steel, P110 oil well casing steel, was cut into suitable sizes and subjected to surface grinding and rust removal. Approximately 300 g of the matrix steel was placed in a high-purity alumina crucible. The crucible and the matrix steel were then placed in the tubular resistance furnace under argon gas protection. The temperature was raised to 1600 °C and maintained for 30 min. Pure cerium blocks (99.9% purity) wrapped in iron foil were inserted into the crucible. Stirring with a molybdenum rod for 5–10 s ensured the homogenization of the steel composition. After 30 min of refining, the reaction reached an equilibrium state. Subsequently, samples were extracted using a negative-pressure quartz tube and immediately quenched in a 5% NaCl solution to prevent reactions during solidification. Based on different Ce addition levels as 0, 0.02%, 0.04%, 0.06%, and 0.08%, the samples were categorized into C0, C1, C2, C3, and C4, respectively. The remaining portion of the matrix steel in the crucible was cooled at a rate of 5 °C/min to 800 °C, and the power supply was then turned off. The remaining sample in the crucible was cooled to room temperature for tensile test and microstructure observation. An argon gas flow rate of 2 L·min^−1^ was maintained throughout the melting process.

### Processing method

**Figure 2:**
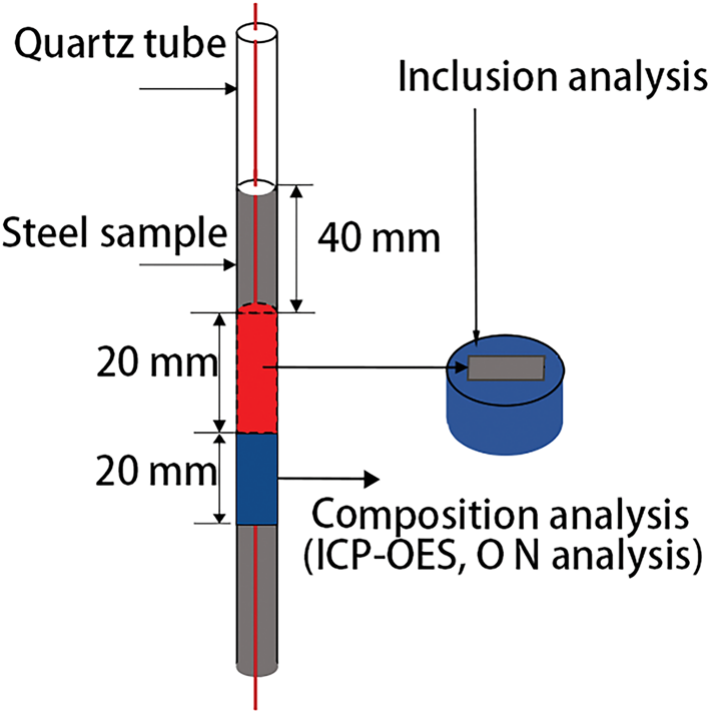
Sample analysis position.

**Figure 3:**
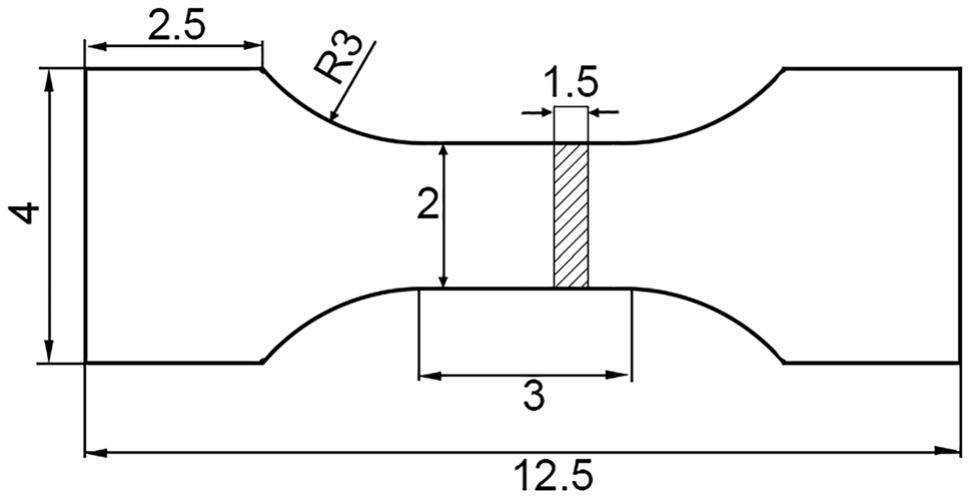
Size of tensile test (mm).

The processing method of each sample is illustrated in Fig. 2. The metal elements of samples, such as Ce and Al content, were determined using inductively coupled plasma atomic emission spectrometry (ICP-AES). O, N, and S contents were analyzed through infrared absorption spectroscopy. The inclusions on the surface of steel sample were detected using a Phenom scanning electron microscope–energy dispersive spectroscopy (SEM–EDS) equipped with the particle X automatic inclusion analysis system. This system enabled automated measurement of inclusion size, distribution, and morphology, statistically analyzing an approximate surface area of 20 mm^2^ for each sample. The remaining samples in the crucible were processed into tensile test specimens using wire-cutting techniques longitudinally along the center of the ingot, the dimensions of the sample for tensile testing are shown in Fig. 3. The tensile properties were tested five times to avoid accidental, the test was at room temperature by an in-situ testing machine (IBTC-5KST, care).

## Result and discussion

### Inclusion analysis

The elemental contents of O, S, N, Ce, and Al in the five groups of samples (C0 to C4) are presented in Table 2. As the rare earth content increases from 0 to 298 ppm, the oxygen content decreases from 58 to 29 ppm. With further increase in cerium content, little change in the oxygen content. As the cerium content increased from 0 to 452 ppm, the sulfur content decreased from 29 to 19 ppm. The four samples exhibit significant fluctuations in aluminum content, which can be attributed to incorporating refractory materials from the crucible into the molten steel.

**Table 2:** Composition of each steel sample 1.

No	Ce addition amount (wt.%)	Sampling time (min)	[Ce]	[Al]	[O]	[S]	[N]
C0	0% Ce	30	0	290	58	29	64
C1	0.02% Ce	30	116	231	41	27	60
C2	0.04% Ce	30	235.2	298	29	28	58
C3	0.06% Ce	30	294.2	513	28	22.3	51
C4	0.08% Ce	30	452	555.8	29	19	47

**Figure 4:**
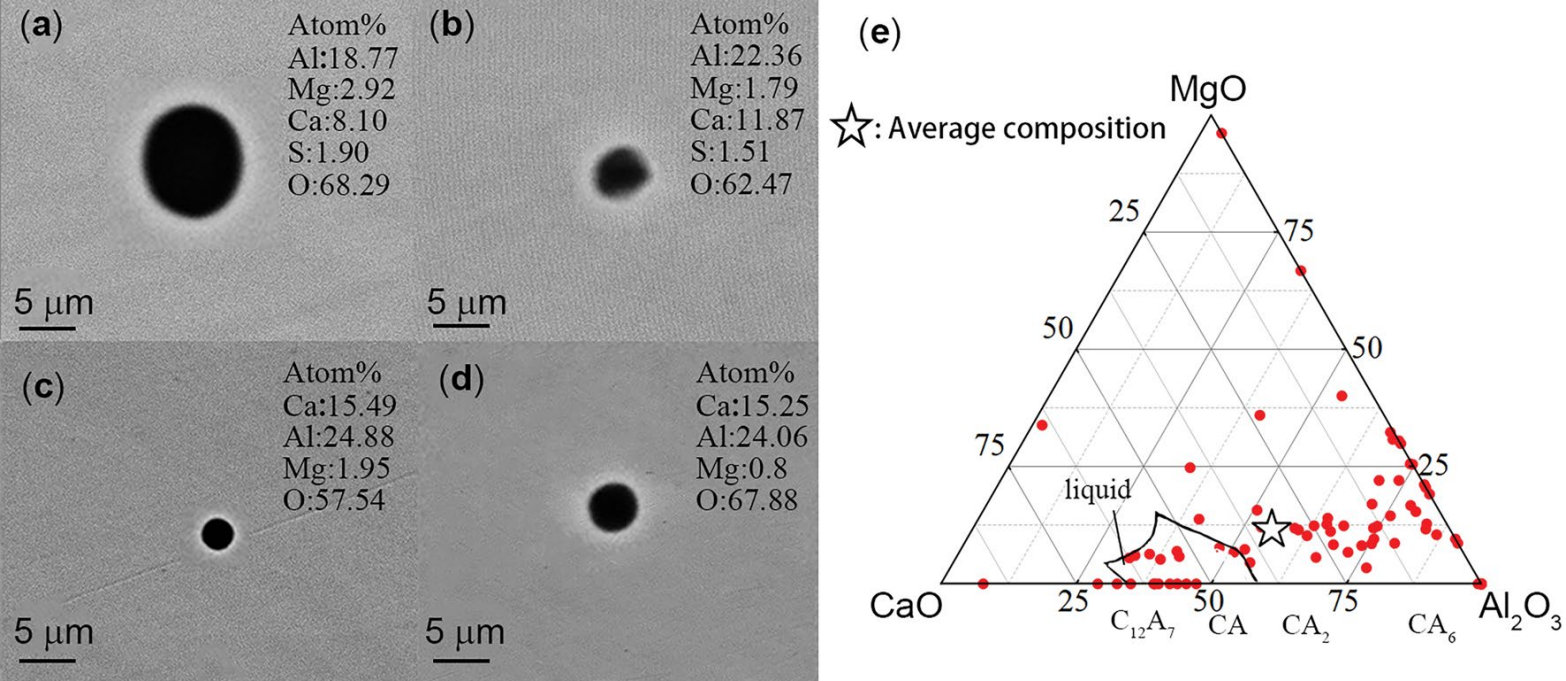
Inclusions in sample C0. (**a**–**d**) The morphology and composition of typical inclusions before Ce treatment. (**e**) The composition distribution of inclusions before Ce treatment.

The selected P110 grade oil casing steel in this experiment undergoes deoxidation with aluminum (Al) during production and is subjected to calcium (Ca) treatment. Therefore, prior to adding cerium (Ce) elements, the predominant inclusions in the steel are calcium aluminate inclusions, along with a small number of complex inclusions such as MgO and CaS. Figure 4a–e show the specific morphologies and compositional distributions of these inclusions. It can be observed that the typical inclusions in Fig. 4a–d exhibit an approximately spherical morphology, indicating that the inclusions are primarily in a liquid state during the refining process. The elements with the highest atomic percentage in these inclusions are O, Ca, and Al, with some inclusions containing small amounts of Mg and S. Figure 4e represents the compositional distribution of the inclusions. The solid line enclosed area represents the liquid phase region at 1600 °C, which was acquired by FTOxid database in Factsage 7.2. It can be observed that some inclusions are aggregated in this region. However, a considerable number of inclusion points are still falling outside this region, closer to the alumina (Al_2_O_3_) region. Furthermore, the average composition of the inclusions is Al_2_O_3_: 57.81, MgO: 11.52, CaO: 32.98, located near CA2 and not within the liquid phase region at 1600 °C. This is attributed to a small amount of Al_2_O_3_ alumina from the alumina crucible that has entered the molten steel, causing the migration of some inclusion components toward the alumina direction.

**Figure 5:**
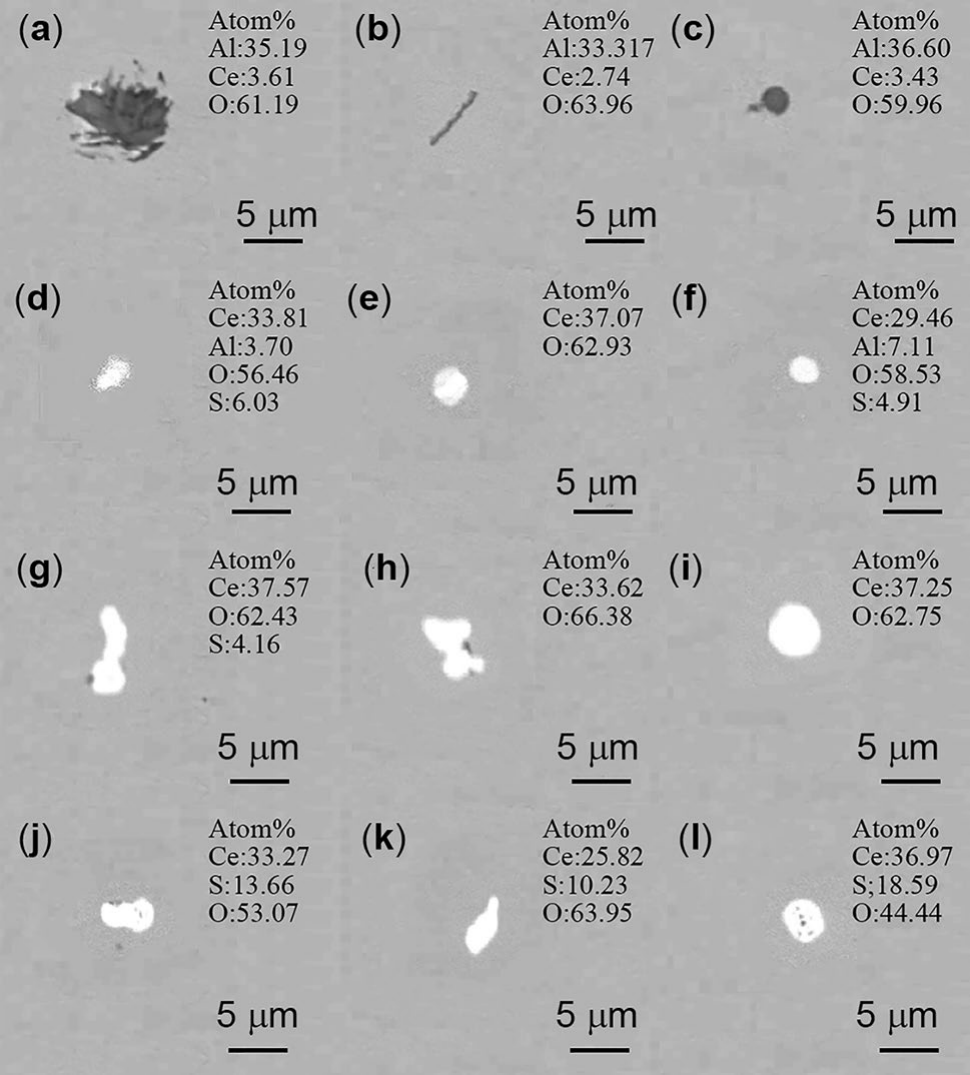
Morphology and composition of typical inclusions in samples C1–C4 (**a**–**c**) C1 (**d**–**f**) C2 (**g**–**i**) C3 (**j**–**l**) C4.

Figure 5 shows the morphologies of typical inclusions in samples C1-C4 after 30 min of treatment with different cerium (Ce) contents. At this stage, the calcium aluminate inclusions in the steel have entirely transformed into Ce-containing inclusions, and the morphology of the inclusions varies with different Ce addition levels. In sample C1, with the addition of 0.01% cerium, the shape of the inclusion’s transitions from predominantly spherical to approximately spherical and elongated. In Fig. 5, as the rare earth addition continues to increase to 0.02–0.08% (samples C2, C3, C4), the color of the inclusion’s changes to bright white, while the shape continues to approximate a sphere. In the typical inclusions of the C1 sample, the elements with the highest atomic percentages are Al and O, accompanied by a small amount of Ce. These inclusions are primarily Al_2_O_3_, with a small amount of CeAlO_3_. In sample C2, C3, and C4, the elements with higher atomic percentages are Ce and O, and the atomic percentage of S also increases with the Ce content increased.

**Figure 6:**
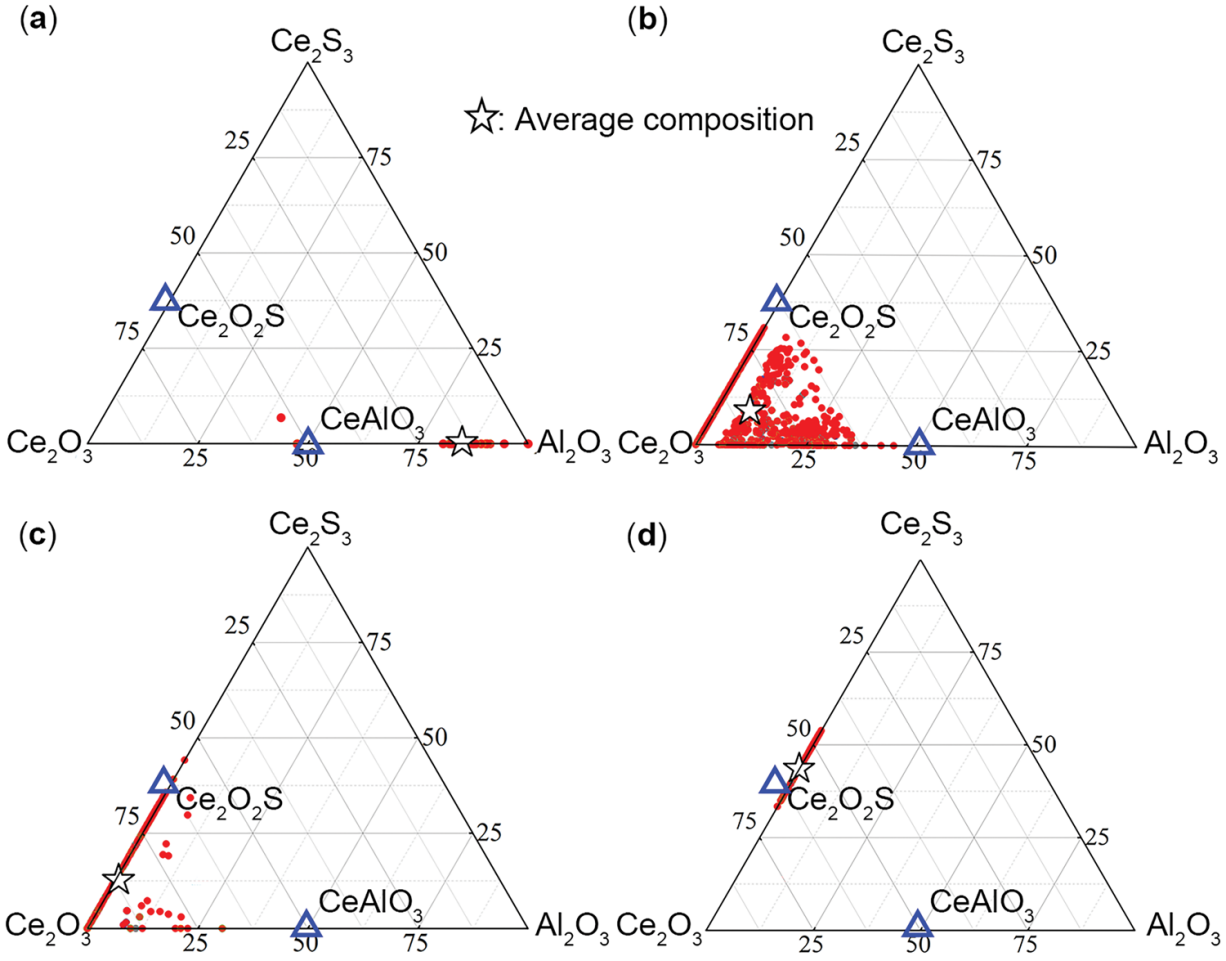
Composition distribution of inclusions (**a**) C1 (**b**) C2 (**c**) C3 (**d**) C4.

The inclusion automatic analysis system was employed to analyze the composition distribution of inclusions in the four samples to obtain a more accurate assessment of the inclusion composition. The average compositions of inclusions were plotted, as shown in Fig. 6. In sample C1, lots of composition points located on the line between CeAlO_3_ and Al_2_O_3_, combined with Table 3; it can be concluded that the primary type of inclusions in sample C1 is Al_2_O_3_–CeAlO_3_. In the same way, the primary type of inclusions in sample C2, C3, and C4 is Ce_2_O_3_(–Al/S), Ce_2_O_3_(–Ce_2_O_2_S), and Ce_2_O_2_S, respectively.

**Table 3:** Average composition of inclusion 1.

No./composition	Al_2_O_3_	CeAlO_3_	Ce_2_O_3_	Ce_2_O_2_S	Ce_2_S_3_
C1	70.04	29.67	0	0	0.29
C2	0	17.92	50.87	31.21	0
C3	0	1.42	60.38	38.2	0
C4	0	1.09	18.93	59.99	20

**Figure 7:**
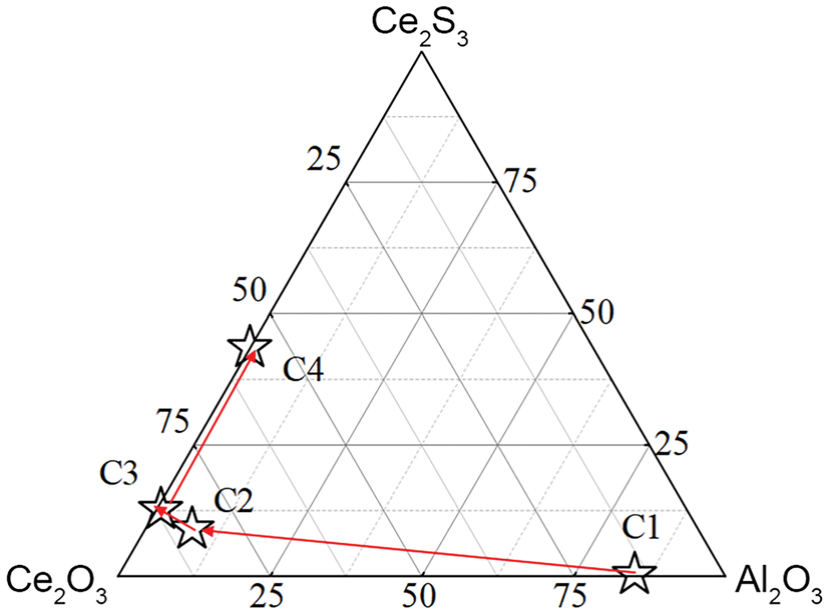
Variation in the average composition of inclusions in the steel with Ce content.

Based on the results obtained from the automatic inclusion analysis, the average composition variations of inclusions in four sample groups with different cerium additions are illustrated in Fig. 7. As the cerium addition increases from 0.02 to 0.04%, the predominant inclusions transform from Al_2_O_3_–CeAlO_3_ inclusions to Ce_2_O_3_–CeAlO_3_ inclusions. At a cerium addition of 0.06%, the inclusions predominantly consist of Ce_2_O_3_. When the cerium addition reaches 0.08%, the inclusions become Ce_2_O_3_–Ce_2_O_2_S composite inclusions—further increasing the cerium addition to 0.08% in the C4 sample results in the dominant inclusion component being Ce_2_O_2_S.

**Figure 8:**
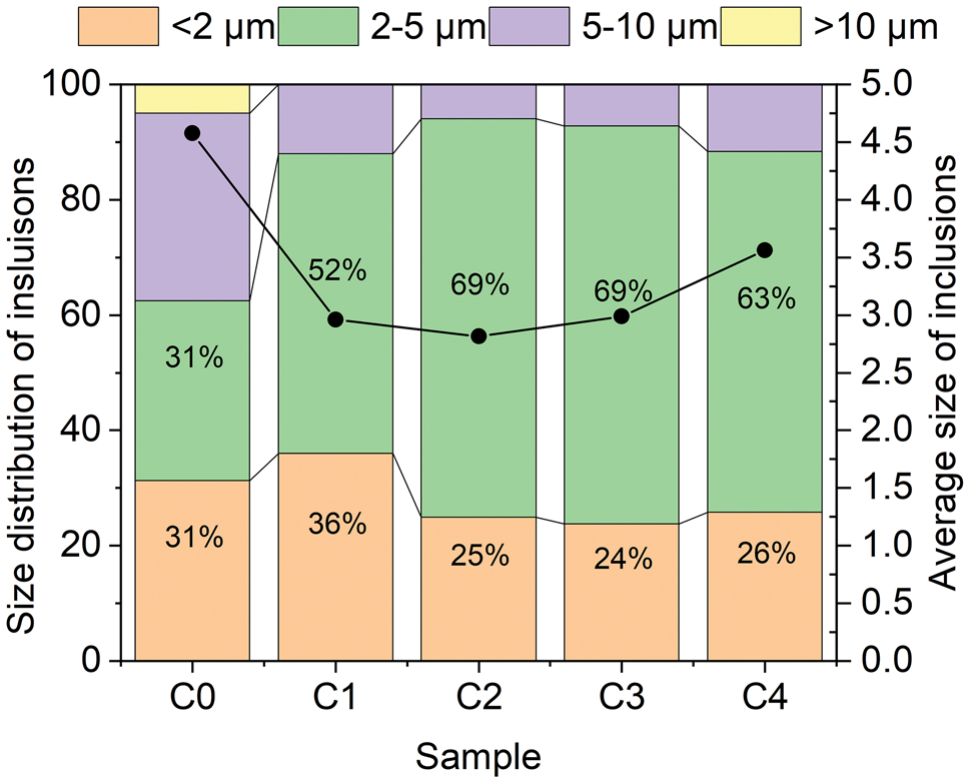
Size distribution of inclusions.

The size distribution of inclusions was counted by SEM to investigate the effect of Ce content on inclusions. The result is shown in Fig. 8. The average sizes of inclusions in sample C0, C1, C2, C3, and C4 are 4.58 μm, 2.96 μm, 2.82 μm, 2.98 μm, and 3.56 μm, respectively. As the amount of Ce added increased, the average size of inclusions first decreased and then increased, and the average size of inclusions was the smallest at 235 ppm cerium content. C0 contains large inclusions with sizes exceeding 10 μm, significantly affecting the performance of steel. It is obviously that C1, C2, C3 has the minimum size, which is mainly due to the lower capillary force between CeO2, CeAlO3, this is consistent with the results of existing studies^27^.

### Thermodynamic calculation

Due to the interaction between rare earth elements and inclusions and dissolved elements in the steel^28^, it is necessary to conduct thermodynamic calculations to speculate on the reaction products and assess the feasibility of the reactions. In the steelmaking process, the temperature of the molten steel is typically controlled at 1873 K (1600 °C). At this temperature, after a sufficiently long reaction time, the reaction rate is often no longer the limiting factor for the occurrence of reactions. Therefore, calculations based on classical thermodynamic principles are reliable for predicting the formation of inclusions in the steel^29,30^. The Gibbs free energy of formation for each inclusion in this study is presented in Table 4. The activity of the product compounds was assumed to be 1 because they were pure substances. The Wagner model calculated the activity of the element dissolved in the liquid steel, as shown in Eq. (1) to Eq. (2); 1% mass steel solution was regarded as the reference state. The interaction coefficients ${\text{e}}^{{\text{i}}}_{{\text{j}}}$ of the common element in P110 grade steel used in the calculation were listed in Table 5. For general reaction a[A] + b[B] = c[C] + d[D], the equilibrium constant K can be expressed as Eq. (3).

1 $$\begin{array}{*{20}c} {a_{i} = f_{i} \cdot w_{i} } \\ \end{array}$$

2 $$\log f_{j} = \mathop \sum \limits_{i}^{n} e_{j}^{i} w_{i}$$

3 $$\log {\text{K}} = {\text{c}}\log a_{{\text{C}}} + {\text{d}}\log a_{{\text{D}}} - {\text{a}}\log a_{{\text{A}}} - {\text{b}}\log a_{{\text{B}}}$$

**Table 4:** Gibbs free energy of inclusions^22,31^.

Inclusions	Reactions	Gibbs free energy J/mol
Al_2_O_3_	2 [Al] + 3 [O] = Al_2_O_3_ (s)	ΔG^θ^ = − 1,203,623 + 386.7T
Ce_2_O_3_	2 [Ce] + 3 [O] = Ce_2_O_3_	ΔG^θ^ = − 714,380 + 179.74T
CeAlO_3_	[Ce] + [Al] + 3 [O] = CeAlO_3_	ΔG^θ^ = − 1,366,460 + 364.3T
CeS	[Ce] + [S] = CeS	ΔG^θ^ = − 422,780 + 121T
Ce_3_S_4_	3 [Ce] + 4 [S] = Ce_3_S_4_	ΔG^θ^ = − 1,495,440 + 439T
Ce_2_O_2_S	2 [Ce] + 2 [O] + [S] = Ce_2_O_2_S	ΔG^θ^ = − 1,353,590 + 332T

**Table 5:** Interaction coefficients of elements in steel^32^.

e^i^_j_	C	Si	Mn	Cu	Ni	V	Ce	S
O	− 0.45	− 0.131	− 0.021	− 0.013	0.006	− 0.3	− 0.57	− 0.133
S	0.11	0.063	− 0.026	− 0.0084	0	− 0.016	− 0.856	− 0.028
Ce	− 0.077	0	0.13	0	0	0	− 0.003	− 39.8
Al	0.091	0.0056	0.012	0.008	0.008	0.06	− 0.43	− 0.03

e^i^_j_	Ca	B	W	N	Nb	Ti	O	Al
O	− 271	− 2.6	− 0.0085	0.057	− 0.14	− 0.6	− 0.2	− 3.9
S	− 100	0.11	0.0097	0.01	− 0.013	− 0.072	− 0.27	0.035
Ce	0	0	0	− 6.599	0	0	− 5.03	− 2.25
Al	− 0.047	0	0	− 0.058	0	0.07	− 6.6	0.045

**Figure 9:**
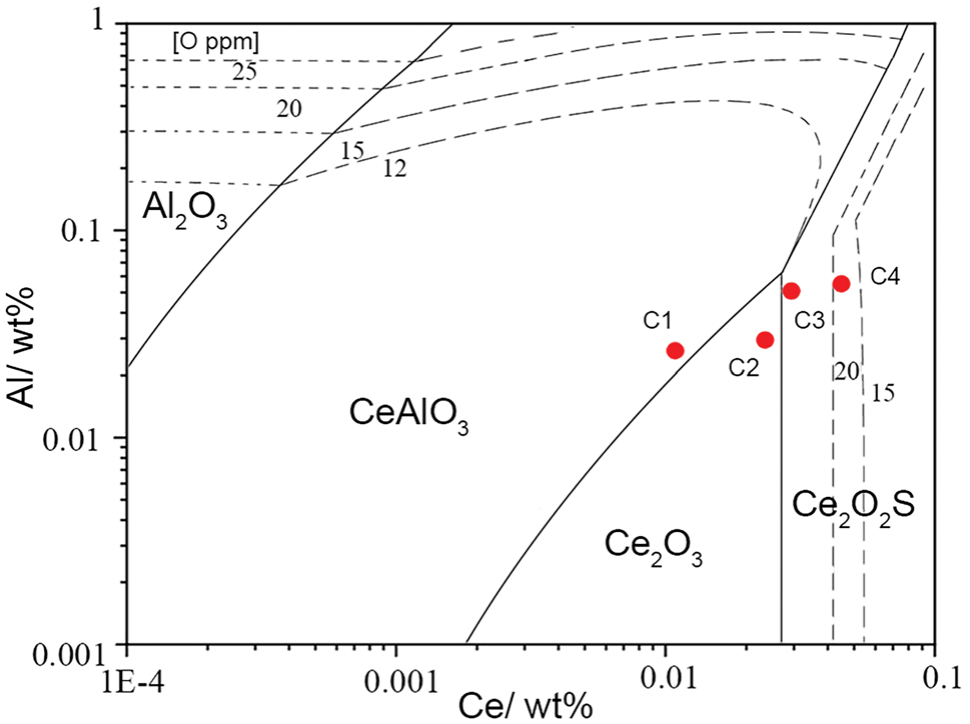
Phase stability diagram of rare earth inclusions in P110 grade casting steel.

In the equations, *a*_*i*_ and *w*_*i*_ represent the activity and concentration of element *i*, respectively. *f*_*i*_ denotes the Henry activity coefficient of element *i*. A and B represent the reactants in a reaction, while C and D represent the products. The coefficients a, b, c, and d represent the stoichiometric coefficients of the respective substances before and after the reaction. When the reaction in the molten steel reaches equilibrium at a temperature of 1873 K, the following Eqs. (4), (5) can be derived from the isothermal equation:

4 $${\Delta G}^{\theta } = - {\text{RTlnK}}$$

5 $$\log {\text{K}} = {{ - {\Delta G}^{{\uptheta }} } \mathord{\left/ {\vphantom {{ - {\Delta G}^{{\uptheta }} } {\left( {{\text{RT}} \cdot {\text{ln}}10} \right)}}} \right. \kern-0pt} {\left( {{\text{RT}} \cdot {\text{ln}}10} \right)}} = {{ - {\Delta G}^{{\uptheta }} } \mathord{\left/ {\vphantom {{ - {\Delta G}^{{\uptheta }} } {35862.597}}} \right. \kern-0pt} {35862.597}}$$

where ΔG^θ^ represents the standard Gibbs free energy of the corresponding reaction, which can be found in Table 4. R is the gas constant (8.314 J/(mol·K)), T is the temperature at equilibrium in Kelvin (K), and K is the equilibrium constant dimensionless number. By combining Eqs. (3), (5), the relationship between the activities of different substances at equilibrium can be obtained. Based on this relationship, a dominance diagram of inclusions in P110-grade oil casing steel is plotted, as shown in Fig. 9.

Figure 9 shows the predominance diagram of Ce–Al–S–O inclusions in oil well casing steel (P110-grade) with sulfur (S) content of 20 ppm. The x-axis and y-axis represent the influence of Ce and Al contents on the inclusions, respectively, while the dashed line represents the oxygen (O) content in ppm. Due to the synergistic effect of high-purity Al_2_O_3_ crucible and added Ce during the experimental process, the Al content in the steel were fluctuates. This diagram was reliable for assessing the inclusions in the actual process, it can be observed that when the Ce content is below 0.001% and the Al content is above 0.02%, Al_2_O_3_ inclusions appear in the system. As the Ce content increases, Al_2_O_3_ inclusions transform into CeAlO_3_ and then further into Ce_2_O_2_S. Only when the Al content is below 0.07%, an increase in Ce content leads to the transformation of CeAlO_3_ into Ce_2_O_3_. When the Al content exceeds 0.07%, an increase in Ce content directly converts CeAlO_3_ inclusions into Ce_2_O_2_S.

Based on the composition analysis results of samples C1, C2, C3, and C4, the red dots in the graph represent the corresponding positions of these four sample groups. The observed inclusions in the samples are compared with the thermodynamic calculation results. In this diagram, C1 is located within the stability range of CeAlO_3_, C2 falls within the Ce_2_O_3_ region, C3 is at the boundary between Ce_2_O_3_ and Ce_2_O_2_S, and C4 is within the Ce_2_O_2_S region. According to the thermodynamic calculation results, after sufficient incubation time at 1873 K (1600 °C), the inclusions that should exist in samples C1, C2, C3, and C4 are CeAlO_3_, Ce_2_O_3_, Ce_2_O_3_–Ce_2_O_2_S, and Ce_2_O_2_S, respectively. The results demonstrate that the observed inclusion composition in the actual experiments is in good agreement with the predicted results from thermodynamic calculations. Therefore, the dominance diagram of P110-grade oil casing steel at 1873 K can effectively predict the end products of steel during the refining process. This is beneficial for predicting and controlling inclusions in actual production processes.

### Tensile strength and microstructure

**Figure 10:**
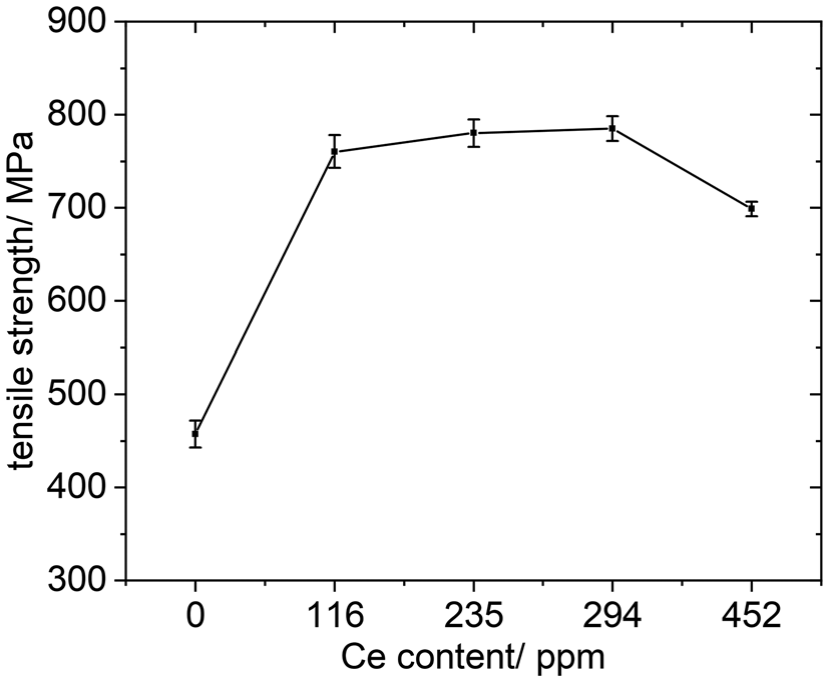
Tensile strength of each sample.

To investigate the influence of different types of inclusions and Ce content on the steel properties, the room temperature tensile properties of four different cerium (Ce) content samples, namely C1, C2, C3, and C4, were tested, as shown in Fig. 10. In this experiment, all samples underwent the same cooling method and were not subjected to subsequent heat treatment or processing. The sole purpose was to compare the performance of P110-grade oil casing steel with different Ce contents and inclusions.

Figure 10 shows that with lower rare earth content, the tensile strength of the steel increased as the rare earth content increased. The highest tensile strength of 784 MPa was achieved when the rare earth content was 294 ppm. However, further increasing the rare earth content decreased tensile strength, suggesting that an excessive amount of rare earth elements could negatively impact the performance. The C3 sample exhibited the highest tensile strength among the four experimental groups.

**Figure 11:**
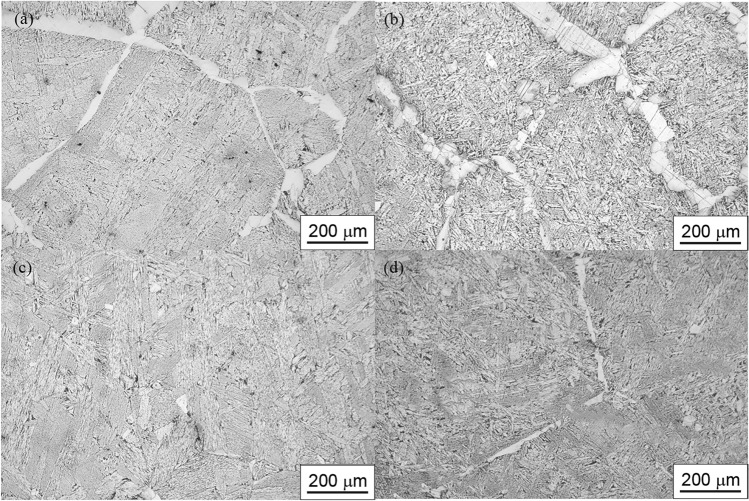
The microstructure of each sample (**a**) C1 (**b**) C2 (**c**) C3 (**d**) C4.

Microstructures of samples C1, C2, C3, and C4 were shown in Fig. 11a–d. In Fig. 11a, it can be observed that the microstructure consists of polygonal ferrite and acicular ferrite. These polygonal ferrites grow along the grain boundaries and exhibits significant anisotropy, making it prone to crack propagation, thereby reducing the strength and toughness of the oil casing steel. Figure 11b also exhibits similar characteristics. As the Ce content further increases to 294 ppm, as shown in Fig. 11c, the polygonal ferrite in sample C3 had disappeared, leaving many fine intragranular acicular ferrites and a small amount of intragranular polygonal ferrites. These ferrites are interlocked and distributed, effectively impeding crack propagation, thereby enhancing the mechanical properties of the oil casing steel. With a further increase in cerium content to 452 ppm, as shown in Fig. 11d, elongated grain boundary ferrites reappear along the austenite grain boundaries.

**Figure 12:**
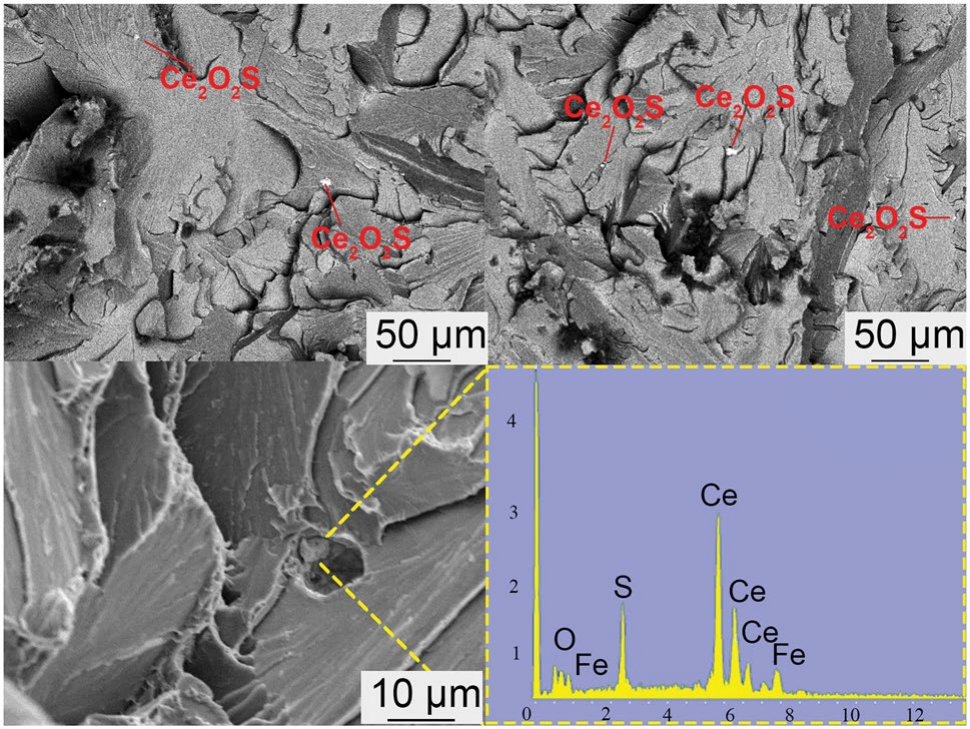
tensile fracture surface of sample C4.

To investigate the significant decrease in performance observed in the C4 sample, SEM–EDS analysis was conducted on the tensile fracture surface, as shown in Fig. 12. The image was captured using a scanning electron microscope (SEM) in backscattered electron (BSE) mode. The black regions represent the low atomic number steel matrix, while the white regions indicate areas containing Ce inclusions. The EDS results indicated the presence of numerous Ce_2_O_2_S inclusions larger than 5 μm at the fracture surface, which suggests that the formation of large-sized Ce_2_O_2_S inclusions at higher Ce content significantly affected the mechanical properties of the steel. The results indicate that an excess of cerium leads to a decline in performance. In future research, it should be considering that the balance between the cost and performance of cerium addition and investigate the effect of cerium content on corrosion performance in simulated oil and gas environments. By addressing these areas, future research can build on the current findings to develop more advanced, high-performance P110-grade oil casing steels, tailored for the demanding conditions of oil and gas extraction.

## Conclusion

This study analyzed the variation of inclusions and their effect on the performance of P110-grade oil casing steel with different Ce contents under laboratory conditions. Based on thermodynamic calculations and experimental data, the following conclusions were drawn:

(1) With increasing rare earth (Ce) content in P110 oil casing steel, the transformation sequence of non-metallic inclusions is as follows: xCaO⋅yAl_2_O_3_ → Al_2_O_3_–CeAlO_3_ → Ce_2_O_3_–CeAlO_3_ → Ce_2_O_3_–Ce_2_O_2_S → Ce_2_O_2_S. As the cerium content increases from 0 to 235 ppm, the size of inclusions gradually decreases from 4.58 to 2.82 μm. However, when the cerium content exceeds 235 ppm, the inclusion size increases with adding cerium.

(2) The Ce–Al–S–O inclusion dominance diagram was plotted based on classical thermodynamics and agreed well with experimental results. With increasing Ce content, Al_2_O_3_ inclusions transform into CeAlO_3_, which further transforms into Ce_2_O_2_S. Ce_2_O_3_ only appears in the system when the Al content is below 0.07%.

(3) Tensile test results indicate that the tensile strength ranking of the four samples is as follows: C3 > C2 > C1 > C4. When the rare earth content in the steel is 294 ppm, the highest tensile strength of 784 MPa is achieved. Further increasing the rare earth content leads to decreased tensile strength, which is attributed to the formation of large-sized Ce_2_O_2_S inclusions and the change of microstructure, which promote crack initiation and extension.

## Data Availability

The datasets used and/or analysed during the current study available from the corresponding author on reasonable request.
